# PRMT5 Inhibitor EPZ015666 Decreases the Viability and Encystment of *Entamoeba invadens*

**DOI:** 10.3390/molecules30010062

**Published:** 2024-12-27

**Authors:** Rigoberto Ortiz-Hernández, Elmer Joel Millán-Casarrubias, Jeni Bolaños, Susana Munguía-Robledo, Carlos Vázquez-Calzada, Elisa Azuara-Licéaga, Jesús Valdés, Mario Alberto Rodríguez

**Affiliations:** 1 Departamento de Infectómica y Patogénesis Molecular, Centro de Investigación y de Estudios Avanzados del Instituto Politécnico Nacional, Mexico City 07360, Mexico; rigoberto.ortiz@cinvestav.mx (R.O.-H.); jbolanosr@outlook.com (J.B.); smunguia@cinvestav.mx (S.M.-R.); carlosv@cinvestav.mx (C.V.-C.); 2 Laboratorio de Sistemas de Diagnóstico y Tratamiento de Cáncer, Unidad Profesional Interdisciplinaria en Ingeniería y Tecnologías Avanzadas del Instituto Politécnico Nacional, Mexico City 07340, Mexico; emillan@cinvestav.mx; 3 Programa de Posgraduados en Ciencias Genómicas, Universidad Autónoma de la Ciudad de México, Mexico City 04510, Mexico; elisa.azuara@uacm.edu.mx; 4 Departamento de Bioquímica, Centro de Investigación y de Estudios Avanzados del Instituto Politécnico Nacional, Mexico City 07360, Mexico; jvaldes@cinvestav.mx

**Keywords:** *Entamoeba invadens*, encystment, symmetric arginine dimethylation, PRMT5

## Abstract

Protein arginine methyltransferase 5 (PRMT5) is an enzyme that produces monomethyl arginine (MMA) and symmetric dimethyl arginine (sDMA), post-translational modifications that regulate several cellular processes, including stage conversion in parasitic protozoans. *Entamoeba histolytica*, the etiologic agent of human amebiasis, has two stages in its life cycle, the trophozoite, which is the replicative form, and the cyst, corresponding to the infective phase. The study of the molecular mechanisms that regulate differentiation in this parasite has been overdue because of a lack of efficient protocols for in vitro encystment. For this reason, *Entamoeba invadens*, a parasite of reptiles, has been used as a differentiation model system for the genus. Here, we demonstrated the presence of sDMA in *E. invadens*, which increases during encystment, and identified the PRMT5 of this microorganism (EiPRMT5). In addition, we performed 3D modeling of this enzyme, as well as its molecular docking with the PRMT5 inhibitor EPZ015666, which predicted the affinity of the drug for the active site of the enzyme. In agreement with these findings, EPZ015666 reduced trophozoite viability and encystment. Therefore, EiPRMT5 is a potential target for inhibiting the spread of amebiasis.

## 1. Introduction

Arginine methylation is a post-translational modification (PTM) that regulates several cellular processes, such as transcription, RNA splicing, protein synthesis, signal transduction, and DNA repair, among others [1]. The terminal guanidinium atoms of the arginine residue can be monomethylated (MMA), asymmetrically dimethylated (aDMA), or symmetrically dimethylated (sDMA) [1]. Different forms of arginine methylation lead to distinct downstream outcomes; for instance, the asymmetric dimethylation of arginine 3 of histone 4 (H4R3me2a) leads to gene activation, whereas its symmetric dimethylation (H4R3me2s) leads to gene repression [2].

Arginine methylation is carried out by enzymes known as protein arginine methyltransferases (PRMTs), which use S-adenosyl-L-methionine (SAM) as a donor of the methyl group; according to their activity, these enzymes are divided into four types [1]: type I produces MMA and aDMA; type II yields MMA and sDMA; type III catalyzes only the formation of MMA; and type IV, which has only been described in yeast and plants, monomethylates the δ-amino of arginine. Mammals have nine members of the PRMT family (PRMT1-PRMT9); six of them are type I (PRMT1, PRMT2, PRMT3, PRMT4, PRMT6, and PRMT8), two are type II (PRMT5 and PRMT9), and one is type III (PRMT7) [1]. Interestingly, dysregulation of arginine methylation is closely associated with cancer progression, suggesting that PRMTs are potential targets for the development of new anticancer drugs [1]. In fact, PRMT5 is emerging as the most promising target for a range of solid and blood cancers [1]. In particular, EPZ015666 is a compound that binds to PRMT5 by competitively inhibiting the binding of the substrate peptide and non-competitively inhibiting SAM binding and has been shown to have antitumor activity [3,4,5].

In protozoan parasites, the types and number of PRMTs vary within each group, but all contain at least one type I and one type II, with higher homology to PRMT1 and PRMT5, respectively [6,7]. Interestingly, PRMT5 has been described as regulating life cycle transitions in some of these microorganisms [8,9]. *Entamoeba histolytica*, the parasite responsible for human amebiasis, infects up to 50 million people worldwide each year, causing 40,000 to 100,000 deaths annually [10]. This parasite has two life cycle forms, trophozoites and cysts. The trophozoite is the replicative form and is responsible for causing disease, but to infect the host, it must differentiate into a cyst. *E. histolytica* contains five PRMTs (EhPRMTs); four of them are type I (EhPRMT1a, EhPRMT1b, EhPRMT1c, and EhPRMTA), and one is type II (EhPRMT5) [6,7]. However, it is not known so far whether these PRMTs participate in the differentiation of this microorganism. The in vitro encystment process of *E. histolytica* has not yet been established. For this reason, *Entamoeba invadens*, a parasite of reptiles that has highly efficient protocols for in vitro encystment, has been used as a model system to study *Entamoeba* differentiation [11].

In this work, we demonstrated that sDMA is produced in *E. invadens* and, interestingly, increases and relocalizes during encystment. Searching for PRMTs in the genome database of this parasite revealed the presence of three type I PRMTs and a single type II PRMT, which shows greater homology with PRMT5. Molecular docking of the 3D model of EiPRMT5 with EPZ015666 showed the affinity of the compound for the active site of the enzyme. Accordingly, the drug reduced trophozoite viability and cyst formation. These results suggest that PRMT5 could be postulated as a potential target for developing new therapeutic strategies to prevent the spread of amebiasis.

## 2. Results

### 2.1. sDMA Occurs in E. invadens and Increases During Encystment

**Figure 1:**
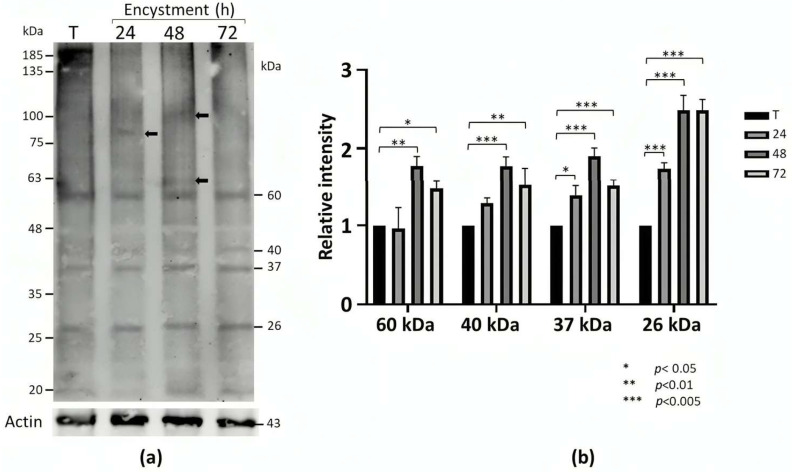
Detection of sDMA in *E. invadens*. (**a**) Extracts of trophozoites (T) and encysting cells at different times (24, 48, and 72 h) were subjected to Western blot assays using an anti-sDMA antibody. Numbers on the right indicate the molecular weights of the main bands detected by the antibody. Arrows indicate specific bands at 24 and 48 h after encystment induction. Actin recognition, used as an internal control, is shown at the bottom. (**b**) The four main bands recognized by the anti-sDMA antibody were analyzed by densitometry, and the data were normalized to those of actin. Data are shown as the mean ± standard deviation of three biological replicates.

**Figure 2:**
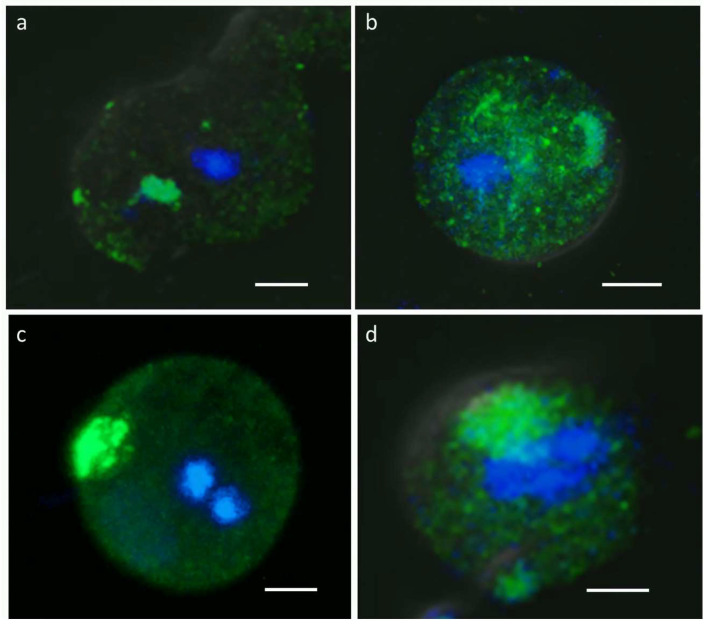
Immunolocalization of sDMA-containing proteins. Three-dimensional reconstruction of the Z-stack scan of trophozoites (**a**) or cells subjected to encystment for 24 h (**b**), 48 h (**c**), and 72 h (**d**) analyzed by confocal immunofluorescence using the anti-sDMA antibody, followed by an ALEXA 488-labeled secondary antibody (green). Nuclei were stained with Hoechst 3342 (blue). Scale bar: 5 µm.

To determine whether symmetric dimethylation of arginine (sDMA) occurs in *E. invadens*, Western blot assays with an anti-sDMA antibody were performed on the extracts of trophozoites and cells subjected to 24, 48, and 72 h of encystment. In these experiments, about 10 protein bands of 20–200 kDa were detected in trophozoite extracts, although recognition was the strongest for the four bands of 26, 37, 40, and 60 kDa (Figure 1a). Interestingly, recognition of proteins with sDMA appeared to increase during encystment, and additional bands of 100, 85, and 62 kDa were also clearly present at 24 or 48 h after encystment induction (Figure 1a, arrows). Densitometric analysis of the four main bands detected by the antibody, using actin recognition as an internal control, confirmed significant augmentation in sDMA (Figure 1b). In trophozoites, by immunofluorescence and confocal microscopy, proteins with sDMA were detected in the nucleus, as small dots located in the cytoplasm and near the plasma membrane, and in some parasites, a fluorescent signal was concentrated in a larger structure of unknown nature (Figure 2a). In cells subjected to encystment for 24 h, fluorescence increased but showed localization similar to that of trophozoites (Figure 2b). Interestingly, sDMA-containing proteins were concentrated at one or two poles of the cells subjected to encystment for 48 and 72 h (Figure 2c,d). These results suggested that symmetrical arginine dimethylation is involved in the encystment of *E. invadens*.

### 2.2. E. invadens Has Only One Type II PRMT

To identify the enzyme catalyzing sDMA in *E. invadens*, a search for putative PRMTs was performed in the genome database of this microorganism. This analysis revealed the presence of four genes (accession numbers EIN_172090, EIN_223690, EIN_398100, and EIN_497480) encoding proteins containing PRMT consensus sequences. The nucleotide sequences range in length from 800 to 1978 bp (Supplementary Table S1), although the start codon of EIN_172090 is missing; consequently, its complete sequence is currently unknown. The EIN_497480 gene does not contain introns, whereas the EIN_398100 and EIN_223690 sequences show the presence of one and two introns, respectively (Supplementary Table S1). The mRNA sizes corresponding to the genes that are complete in the database range from 966 to 1827 nucleotides, encoding proteins with 321 to 608 amino acids with estimated molecular weights of 37 to 69.8 kDa and isoelectric points of 5.10 to 5.57 (Supplementary Table S1).

**Figure 3:**
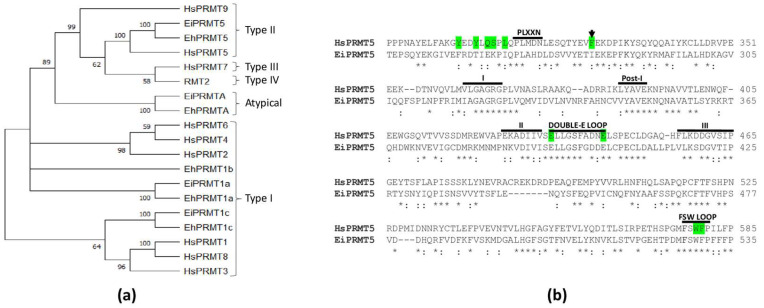
Phylogenetic analysis of EiPRMTs and alignment of human and *E. invadens* PRMT5. (**a**) The predicted amino acid sequences of *E. invadens* PRMTs (EiPRMTs) were aligned with those of human (HsPRMTs), *E. histolytica* (EhPRMTs), and yeast type IV (RMT2), and the data were subjected to phylogenetic analysis by the neighbor-joining method using MEGA11 [12]. Proteins used for this analysis were as follows: human: HsPRMT1 (NP_938074.2), HsPRMT2 (NP_001526.2), HsPRMT3 (NP_005779.1), HsPRMT4/CARM1 (NP_954592.1), HsPRMT5 (NP_006100.2), HsPRMT6 (NP_060607.2), HsPRMT7 (NP_061896.1), HsPRMT8 (NP_062828.3), and HsPRMT9 (NP_612373.2); *S. cereviciae*: RMT2 (NP_010753.1); *E. histolytica*: EhPRMT1a (EHI_105780), EhPRMT1b (EHI_152460), EhPRMT1c (EHI_202470), EhPRMTA (EHI_159180), and EhPRMT5 (EHI_158560); *E. invadens*: EiPRMT1a (EIN_497480), EiPRMT1c (EIN_398100), EiPRMTA (EIN_172090), and EiPRMT5 (EIN_223690). Numbers at branch nodes indicate confidence percentages of the tree topology from the bootstrap analysis of 1000 replicates. (**b**) Alignment of human and *E. invadens* PRMT5, showing that the parasite protein contains the characteristic sequences of PRMTs (domain I; Post-I, II, and III; and the double-E domain), as well as typical sequences of the PRMT5 family, such as the FSW loop and the PLXXN motif. (*) Identical residues; (:) Conservative substitutions. Residues shaded in green correspond to those of HsPRMT5 involved in the binding and catalysis of a histone-H4-derived substrate peptide [13]. The arrow indicates residue F327, which determines sDMA production in HsPRMT5 [14].

A phylogenetic analysis comparing the amino acid sequences of *E. invadens*, *E. histolytica*, and human PRMTs, as well as the type IV PRMT of yeast (RMT2), clustered two of the *E. invadens* PRMTs with type I enzymes; another was related to the atypical PRMT of *E. histolytica* (EhPRMTA); and the last was grouped within the type II family and more closely associated with PRMT5 (Figure 3a). Remarkably, this analysis exposed that *E. invadens* proteins are closely related to EhPRMT1a, EhPRMT1c, EhPRMTA, and EhPRMT5, where the amino acid sequence identity of each EiPRMT with its respective EhPRMT ranges between 46.3 and 83.7% (Supplementary Table S2). Due to this relationship, the *E. invadens* proteins were named EiPRMT1a (EIN_497480), EiPRMT1c (EIN_398100), EiPRMTA (EIN_172090), and EiPRMT5 (EIN_223690), and we suggest that they might have similar functions in both species.

### 2.3. EiPRMT5 Retains the Characteristic Domains of the PRMT5 Family

The amino acid sequence of EIN_223690 was similar to that of PRMT5 from different organisms, including the human protein (HsPRMT5), with which it showed 32.98% identity, supporting the hypothesis that it corresponds to the PRMT5 of *E. invadens* (EiPRMT5). Furthermore, the sequence alignment of EiPRMT5 and HsPRMT5 showed that the *E. invadens* protein contains typical PRMT motifs [15], such as domain I (residues 321–325), post-I (residues 348–350), domain II (residues 386–391), and domain III (residues 417–425), as well as the double-E loop (residues 393–403) (Figure 3b). In addition, it possessed sequences described as specific for PRMT5 [15], such as the FSW loop (residues 527–530); the PLXXN sequence (residues 268–272), although the asparagine residue is replaced by aspartate (Figure 3b); and an N-terminal TIM barrel domain. Furthermore, this alignment revealed that of the 10 amino acid residues of HsPRMT5 involved in substrate binding and catalysis [13], EiPRMT5 has 4 identical ones and another 4 are present as conservative substitutions (Figure 3b), supporting the hypothesis that it belongs to the PRMT5 family.

### 2.4. The 3D Model of EiPRMT5 Showed Homology with the X. laevis PRMT5 Crystal

**Figure 4:**
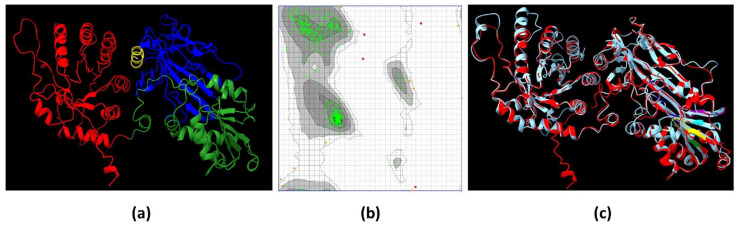
A 3D model of EiPRMT5. (**a**) Ribbon representation of the EiPRMT5 structure predicted by I-TASSER. Red: N-terminal TIM barrel domain. Green: Rossman fold domain. Blue: C-terminal β-barrel domain. Yellow: dimerization arm. (**b**) A Ramachandran plot of the model showed that 97.52% of the residues are located in the most favored regions. (**c**) Merge of the EiPRMT5 model (red) and the *X. laevis* PRMT5 crystal (Cian).

The structural conformation of EiPRMT5 (Figure 4a) was predicted by the I-TASSER server considering the crystal structure of *Xenopus laevis* PRMT5 (PDB: 4G56) as a template. Evaluation of this model was performed through a Ramachandran plot, which showed that 97.52% of the residues are located in the most favored regions, 1.72% in the allowed regions, and 0.76% in the disallowed regions (Figure 4b), data that meet the standard criterion for high-quality protein structures. The overall folding of the EiPRMT5 model displayed (i) an N-terminal TIM barrel domain (residues 9–246), which in the human enzyme binds to the cofactor known as methylosoma protein 50 (MEP50); (ii) a Rossman fold domain (residues 247–426), which is responsible for substrate binding and catalysis; and (iii) a C-terminal β-barrel domain (residues 438–447), involved in dimerization [16] (Figure 4a). When the 3D model of EiPRMT5 was aligned with the crystal structures of PRMT5 from *X. laevis* (Figure 4c) and humans (PDB: 4GQB), the identity of 33 and 29% was found, respectively.

### 2.5. Molecular Docking Predicts the Binding of EPZ015666 to the Active Site of EiPRMT5

**Figure 5:**
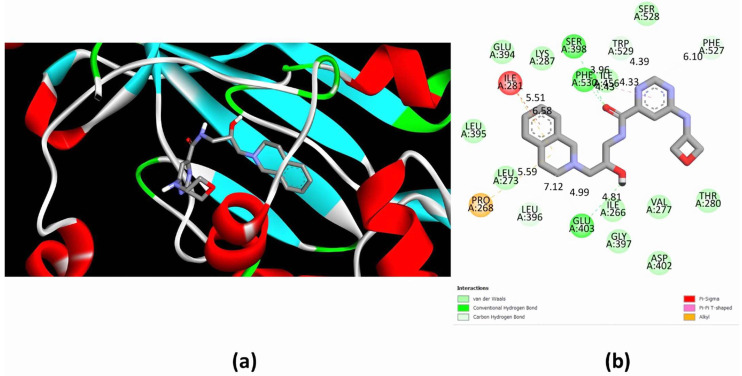
Interactions between EiPRMT5 and EPZ015666. Blinded molecular docking studies between the EiPRMT5 enzyme and the EPZ015666 inhibitor were performed using Autodock 4.2. (**a**) The ligand-binding region in the 3D model of EiPRMT5. (**b**) A 2D model showing the amino acids of EiPRMT5 with which EPZ015666 interacts. The nature of protein–ligand interactions is shown with different colored legends.

To analyze the potential binding of EPZ015666, a specific inhibitor of mammalian PRMT5 [5], to EiPRMT5, we performed blind molecular docking studies using AutoDock 4.2 (Figure 5a). This analysis predicted that the drug binds to the amebic enzyme at residues that, according to the alignment with HsPRMT5 (Figure 3), comprise the peptide-binding site and the pocket occupied by the side chain of the arginine substrate, similarly to what happens with the human enzyme [5,17] (Table 1). This analysis estimated that the drug attaches to EiPRMT5 with a free energy of ligand–receptor binding (ΔG_b_°) = −6.05 kcal/mol. The predicted molecular interactions (Figure 5b) occurred between the tetrahydroisoquinoline (THIQ) and pyrimidine rings of EPZ015666 and residues located mainly in the double-E and FSW loops, which are potentially involved in methyl group transfer. These interactions included three hydrogen bonds with residues S398, E403, and F530, (lengths of 3.96, 4.81, and 4.43 Å, respectively) and four carbon–hydrogen bonds with residues L396, E403, F527, and W529 (lengths of 7.12, 4.99. 6.10, and 4.39 Å), as well as several hydrophobic interactions, such as a Pi–sigma interaction (residue I281 with 5.51 Å), a Pi–Pi interaction (residue F530 with a distance of 4.33 Å), and two Pi–alkyl interactions (residues P268 and I281 with distances of 5.39 and 6.58 Å) (Figure 5b). In addition, several residues were involved in drug binding via Van der Waals forces (Figure 5b), including E394, and S528, located in double-E and FSW loops, respectively.

**Figure 6:**
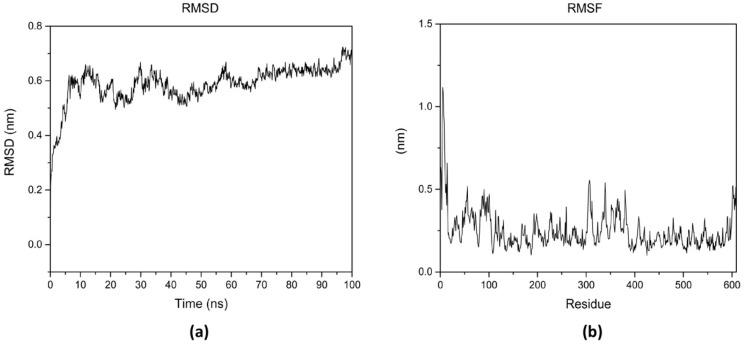
Molecular dynamic simulation of the EiPRMT5–EPZ015666 complex. The complex was subjected to molecular dynamics simulation for 100 ns. (**a**) RMSD plot. (**b**) RMSF plot.

To analyze the stability of the EiPRMT5–EPZ015666 complex, it was subjected to molecular dynamic simulation. The RMSD plot showed that the protein folds after 12 ns of simulation, reaching a deviation of 0.6 nm, and remained within a narrow range for the rest of the 100 ns simulation time (Figure 6a). The RMSF plot did not show large fluctuations in the protein residues and revealed that residues potentially constituting the active site (residues 393–403 and 527–530) exhibit less flexibility (Figure 6b). These results indicate the conformational stability of the complex during the simulation time.

### 2.6. EPZ015666 Affects the Viability and Encystment of E. invadens

**Figure 7:**
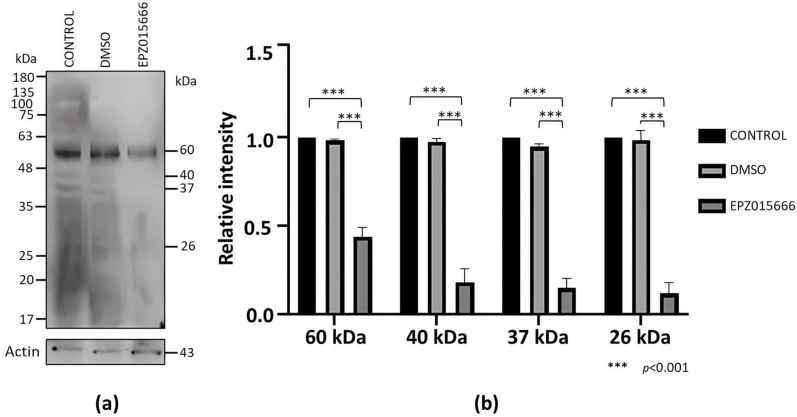
sDMA level in trophozoites cultured in the presence of EPZ015666. (**a**) Extracts of trophozoites cultured for 72 h in a normal medium (control) or the presence of DMSO (vehicle) or EPZ015666 *IC*_20_ were subjected to Western blot assays using an anti-sDMA antibody. Numbers on the right indicate the molecular weights of the main bands detected by the antibody. Actin recognition is shown at the bottom. (**b**) The four main bands recognized by the anti-sDMA antibody were analyzed by densitometry, and the data were normalized with respect to those of actin. Data are shown as the mean ± standard error of three biological replicates.

To analyze the impact of EPZ015666 on the viability of *E. invadens*, we incubated trophozoite cultures in the presence of different concentrations of the drug for 72 h. In this assay, we observed a dose–response effect, with an *IC*_50_ of 96.6 ± 6 μM. Furthermore, Western blot assays showed that sDMA recognition decreased in trophozoites treated with the drug (*IC*_20_ = 20 mM) for 72 h compared to controls, while actin detection, used as an internal control, was similar under all conditions (Figure 7a). Through densitometric analysis of the four main sDMA bands, normalized to the actin content, a significant decrease in this PTM upon treatment with EPZ015666 was confirmed (Figure 7b).

**Figure 8:**
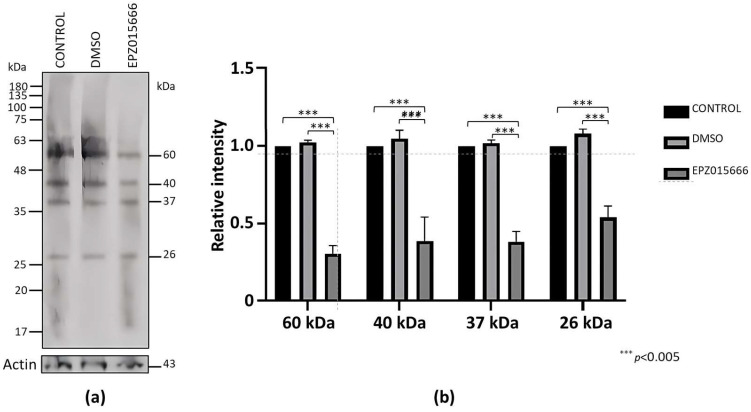
sDMA level during encystment in the presence of EPZ015666. (**a**) Trophozoites were submitted to encystment in the presence of EPZ015666 *IC*_20_ for 72 h. Extracts were then subjected to Western blot assays using an anti-sDMA antibody. Numbers on the right indicate the molecular weights of the main bands detected by the antibody. Actin recognition is shown at the bottom. (**b**) The four main bands recognized by the anti-sDMA antibody were analyzed by densitometry, and the data were normalized with respect to those of actin. Data are shown as the mean ± standard deviation of three biological replicates.

In contrast, Western blot on extracts of cells submitted to encystment and treated with EPZ015666 *IC*_20_ for 72 h also showed a decrease in recognition by the anti-sDMA antibody (Figure 8a). Densitometric analysis of the four main sDMA bands, normalized to the actin content, demonstrated a significant reduction in sDMA in the presence of EPZ015666 (Figure 8b).

**Figure 9:**
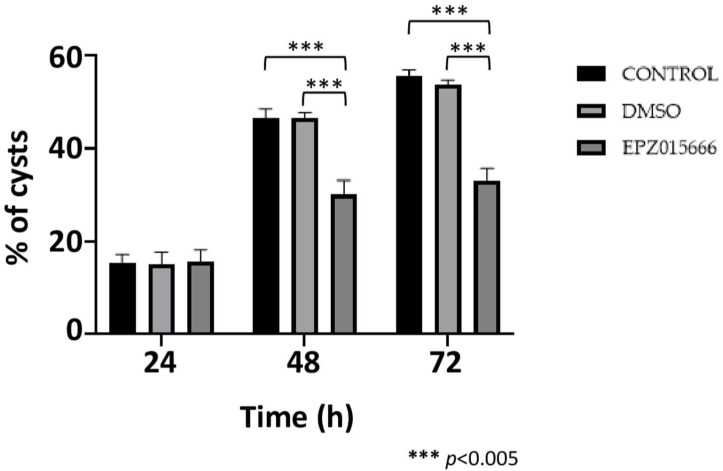
Encystment in the presence of EPZ015666. Trophozoites were submitted to encystment in the presence of EPZ015666 *IC*_20_. The percentage of Sarkosyl-resistant cells was then determined at 24, 48, and 72 h. Data are shown as the mean ± standard deviation of three biological replicates.

Finally, encystment efficiency in the presence of EPZ015666 *IC*_20_ was investigated. In this experiment, cyst development at 24 h post-induction was not affected by the drug; however, at 48 and 72 h, cyst formation in the presence of EPZ015666 decreased by approximately 35 and 40%, respectively, while the DMSO vehicle had no effect (Figure 9). The results confirm that symmetric arginine dimethylation, catalyzed by EiPRMT5, is involved in this *E. invadens* differentiation event.

## 3. Discussion

sDMA is catalyzed by type II PRMTs, and the main enzyme of this type found in all eukaryotic species studied is PRMT5. As we know, PRMT5 is the only type II PRMT described for protozoan parasites [6,7]. Interestingly, it has been described that PRMT5 plays important roles in the invasion of hosts and/or in the life cycles of some of these microorganisms. For instance, in *Plasmodium falciparum* and *Leishmania braziliensis*, PRMT5 disruption decreases parasite invasion [18,19]. In *Toxoplasma gondii*, PRMT5 shows changes in expression and cellular localization in tachyzoite and bradyzoite stages, suggesting that it may be involved in tachyzoite–bradyzoite transformation [8]. In the case of the opportunist parasite *Acantamoeba castellanii*, expression levels of PRMT5 increase during encystment, and PRMT5-knockdown parasites fail to form mature cysts [9]. These results make PRMT5 a potential target for novel anti-parasitic drugs; indeed, it has been reported that onametostat, a *P. falciparum* PRMT5 inhibitor, exhibits antimalarial activity [20].

Our in silico analysis showed that like other protozoan parasites, members of the *Entamoeba* genus have only one type II PRMT, which belongs to the PRMT5 family; however, the role of this enzyme in the biology of these organisms is unknown so far, although we suggest that it might participate in pathogenicity and/or stage conversion. Here, we demonstrated that sDMA is upregulated and relocalized during encystment induction of *E. invadens*, supporting the hypothesis that EiPRMT5 plays an important role in *Entamoeba* differentiation. PRMT5 from higher eukaryotes forms complexes with MEP50, a seven-bladed WD40 repeat β-propeller protein, which enhances the methyltransferase activity of PRMT5 by improving substrate binding [21]. In these organisms, PRMT5 forms a hetero-octameric complex composed of four PRMT5 proteins and four MEP50 proteins [13,21]. Searching the AmoebaDB genome database, we did not identify clear homologs of MEP50 in *E. invadens*, although it has several WD-repeat-containing proteins, and some of them could perform a function similar to that of MEP50. Alternatively, EiPRMT5 might not require another protein to regulate its activity, as has been shown for PRMT5 from *Trypanosoma brucei* [22].

EPZ015666 is a selective PRMT5 inhibitor that binds to the peptide-binding site, giving the compound a peptide-competitive and SAM-uncompetitive characteristic, and its antitumor role has been tested in several cancer cell lines [4,23,24,25]. The inhibitor binds to HsPRMT5 via hydrogen bonds with F580 and E444; furthermore, the tetrahydroisoquinoline (THIQ) ring of EPZ015666 forms a water-mediated interaction with E435 and a Pi–Pi stacked interaction with F327 [5], a residue that directs the symmetric dimethylation of arginine [14]. In concordance, our molecular docking analysis with EiPRMT5 predicted that the inhibitor forms hydrogen bonds with E403 and F530. It also interacts with E394 via Van der Waals forces and forms Pi–sigma and pi–alkyl interactions with I281, suggesting that this compound may also inhibit EiPRMT5. The results predicted some hydrogen bonds over long distances, and it is known that this kind of interaction may be possible if the distance is not greater than 4 Å. However, our study was performed in a rigid system, so in a system closer to reality, these distances would possibly fluctuate closer to (or further from) the ligand.

Incubation of trophozoites or cells undergoing encystment in the presence of EPZ015666 diminishes sDMA production, confirming the molecular docking prediction. However, the *IC*_50_ for trophozoite viability (96.6 μM) is much higher than that for mammalian cells, which varies from 22 nM [5] to approximately 10 μM [23]. This discrepancy could be due to differences in plasma membrane permeability, changes in some residues involved in substrate binding, or the possible absence of a protein that increases the affinity for the substrate, as occurs with MEP50 for PRMT5 from higher eukaryotes [26]. Alternatively, sDMA could not be important for trophozoite viability, although the many pathways regulated by PRMT5/sDMA in other organisms suggest that this possibility is unlikely. Therefore, it will be necessary to test other currently studied PRMT5 inhibitors or chemical modifications of EPZ015666 to specifically reduce amebic viability. Furthermore, it would be important to identify the target proteins of EiPRMT5 and their role in parasite biology. Nevertheless, incubation of cells undergoing encystment with *IC*_20_ of EPZ015666 reduces cyst formation by up to 40%, supporting the idea that sDMA is involved in encystment. Therefore, our results suggest that PRMT5 could be considered a potential target for the development of novel therapeutic strategies against amebiasis.

## 4. Materials and Methods

### 4.1. Culture of E. invadens Trophozoites and In Vitro Encystment

*E. invadens* trophozoites (IP-1 strain) were maintained in LYI-S-2 medium at 25 °C and harvested, as described [27]. To induce encystment, trophozoites were transferred at a final concentration of 5 × 10^5^ cells/mL to low-glucose (LG) medium (LYI-S-2 medium diluted to 47% without glucose and 5% bovine serum) [28], and cells were collected at 24, 48, and 72 h. To confirm encystation, cells were incubated in 0.05% Sarkosyl for 30 min at room temperature. In addition, some samples of detergent-resistant cells were stained with 1% Calcofluor white (CFW) for 30 min at room temperature and visualized under a fluorescence microscope (Olympus BX41, Olympus Corporation of the Americas, Breinigsville, PA, USA).

### 4.2. Western Blot Assays

To determine the presence of sDMA in *E. invadens*, extracts of trophozoites and cells subjected to encystment were separated by 10% SDS-PAGE, and proteins were transferred to nitrocellulose membranes and probed with an anti-sDMA antibody (Cell Signaling Technology, Danvers, MA, USA) (dilution 1:2000). The membranes were then incubated with a peroxidase-coupled secondary antibody (Jackson ImmunoResearch Laboratories, West Grove, PA, USA) (1:10,000 dilution). Reactions were developed by chemiluminescence (ECL Plus, Amersham Technology, Middlesex, England) following the manufacturer’s recommendations. To evaluate semi-quantitively the symmetric dimethylation of arginine, the same membranes were probed with an anti-actin antibody (Santa Cruz Biotechnology, Santa Cruz, CA, USA) (1:3000 dilution), the four main bands recognized by anti-sDMA were analyzed by densitometry, and the data were normalized to that of the band detected by anti-actin. The sDMA level of each band in trophozoites was arbitrarily taken as 1.

### 4.3. Immunofluorescence and Confocal Microscopy

Trophozoites and cells subjected to encystment were fixed and permeabilized with 100% cold methanol for 5 min. Non-specific binding sites were blocked with 10% fetal bovine serum in PBS; next, samples were incubated overnight at 4 °C with the anti-sDMA antibody (dilution 1:100) and subsequently with a secondary antibody coupled to Alexa448 (Jackson ImmunoResearch Laboratories, dilution 1:200). Finally, nuclei were counterstained with Hoechst 33342 (Thermo Fisher Scientific, Waltham, MA, USA), and the cells were observed through a confocal microscope (Carl Zeiss LSM 900, Calrl Zeiss Scientific, Oberkochen, Germany) using ZEN 3.6 software. Observations were performed on 13 planes from top to bottom of each sample; the distance between scanning planes was 1 μm.

### 4.4. Identification of E. invadens PRMTs and In Silico Characterization of EiPRMT5

The search for putative *E. invadens* PRMTs (EiPRMTs) was performed in AmoebaDB (https://amoebadb.org; accessed on 27 February 2023) using the PRMT consensus amino acid sequence as a query. The predicted gene and protein sequences were analyzed in silico using the software deposited on the NCBI Homepage (https://www.ncbi.nlm.nih.gov; accessed on 2 March 2023) and Expasy Bioinformatics Resource (https://web.expasy.org; accessed on 3 March 2023).

The amino acid sequences of EiPRMTs were compared to those of human and *E. histolytica* PRMTs, as well as to yeast type IV PRMT (RMT2), using Clustal Omega software (https://www.ebi.ac.uk/jdispatcher/msa/clustalo; accessed on 6 March 2023). Phylogenetic analyses were carried out using the neighbor-joining method implemented in the MEGA11 software package [12]. Bootstrapping was performed for 1000 replicates.

### 4.5. EiPRMT5 Modeling

The 3D structure of EiPRMT5 was predicted by the I-TASSER server (https://zhanggroup.org/I-TASSER, accessed on 15 March 2023) using the homologous protein from Xenopus laevis (PDB: 4G56) as a template. The predicted structure was then evaluated using the Ramachandran plot on the Molprobity server (https://molprobity.biochem.duke.edu; accessed on 20 April 2023). Protein structure alignment between the EiPRMT5 model and the X. laevis PRMT5 crystal (PDB: 4G56) was carried out with CHIMERA X software (https://www.cgl.ucsf.edu/chimera; accessed on 5 May 2023).

### 4.6. Molecular Docking Analysis of the Interaction Between EiPRMT5 and EPZ015666

The 2D structure of EPZ015666 was obtained with BIOVIA Draw 2022 and then converted to 3D mol format in Avogadro software (https://sourceforge.net/projects/avogadro; accessed on 22 January 2024). Its geometry was then optimized using Gauss View 5.0 software [29] and the AM1 semi-empiric method. The EiPRMT5 model was modified using the BIOVIA Discovery Studio Visualizer and AutoDock Tools 1.5.6 by incorporating charges and polar hydrogen atoms.

For molecular docking studies, we used the AutoDock Tools suite version 1.5.7 (https://autodocksuite.scripps.edu, accessed on 30 January 2024). Blind docking was conducted on the entire protein with 10,000,000 energy evaluations using a Lamarckian genetic algorithm, and a total of 100 runs were executed. The protein was enclosed in a grid box of dimensions 126 × 126 × 126 Å, maintaining a spacing of 0.375 Å. The results obtained were visualized in BIOVIA Discovery Studio Visualizer software. For evaluation of the stability of the EiPRMT5–EPZ015666 complex, two main parameters were considered, root mean square deviation (RMSD) and root mean square fluctuation (RMSF). The protein–ligand complex with the best docking free energy obtained by the Autodock Tools suite was prepared for molecular dynamics using GROMACS [30]. The ligand topology and parameter files were made using the CGenFF web server (https://app.cgenff.com/homepage, accessed on 22 January 2024), whereas the topology file of PRMT5 was generated using the pdb2gmx command and the CHARMM36 all-atom force field. The complex was subsequently solvated in a cubic box using the TIP3P water model and placed in SPC216, maintaining a setback distance of 1.0 nm between the complex and the box’s edge. This system was neutralized by adding sodium or chloride ions, depending on the total charges. The system was then minimized and equilibrated for 100 ps under constant NVT and NPT, with a temperature of 300 K and pressure of 1.0 bar. Finally, the complex underwent a manufacturing process lasting 100 ns, during which time, the RMSD and RMSF of the protein backbone were measured. The procured results were then evaluated against the results obtained from the MDS of the unbound PRMT5. The approximate number of frames per simulation was 5000. The RMSD parameter was evaluated using the WebGRO server (https://simlab.uams.edu, accessed on 1 December 2024). The CABS-flex server version 2.0 (https://biocomp.chem.uw.edu.pl/lCABSfex2, accessed on 4 December 2024) was used to evaluate the RMSF. The number of cycles was set to 50, and the cycles between trajectory frames were set to 50. The other parameters were set to default values. Furthermore, molecular dynamic simulation was also performed by means of imods [31] using default parameters.

### 4.7. Effect of EPZ015666 on E. invadens Trophozoites

To analyze the effect of EPZ015666 on the viability of trophozoites, 2 × 10^5^ cells were seeded in LYI-S-2 medium in the presence of different concentrations of this drug. After 72 h, the number and viability of trophozoites were determined by trypan blue dye exclusion in a hematocytometer. As controls, cells were cultured in the absence of the drug or the presence of DMSO. *IC*_20_ and *IC*_50_ values were obtained using Graphpad Prism 10 (Graphpad Software Inc., Boston, MA, USA) by constructing a dose–response curve.

To examine the effect of EPZ015666 on sDMA in *E. invadens* trophozoites, cells were cultured in LYS-S-2 medium in the presence of *IC_20_* and IC_50_ of the drug or the presence of DMSO (vehicle) for 72 h. Next, the relative level of sDMA at each time was analyzed by Western blot, as described earlier. The relative level of sDMA in trophozoites in the absence of the drug was arbitrarily taken as the unit.

### 4.8. Effect of EPZ015666 on Encystment

Extracts from cells submitted to encystment for 72 h in the absence or presence of DMSO or *IC*_20_ of EPZ015666 were analyzed by Western blotting, and the sDMA level was determined, as shown earlier, arbitrarily taking the relative level of sDMA in the absence of EPZ015666 as 1.

Finally, to analyze the effect of EPZ015666 on cyst formation efficiency, trophozoites undergoing encystment were incubated with *IC*_20_ of the drug, and the number of Sarkosyl-resistant cells was determined at 24, 48, and 72 h.

### 4.9. Statistics

Data are shown as the mean ± standard deviation of three to four biological replicates. Statistical analysis was performed using GraphPad Prism 10.

## 5. Conclusions

The results of this study suggest that PRMT5 is a potential target for the development of new therapeutic strategies against the spread of amebiasis.

## Figures and Tables

**Table 1:** Residues of HsPRMT5 and EiPRMT5 involved in EPZ015666 binding.

Human PRMT5 ^a^	*E. invadens* PRMT5
L319	L273
T323	V277
F327	I281
E435	E344
L437	L396
---	S398
E444	E403
W579	W529
F582	F530

^a^ Zhu et al. [17].

## Data Availability

Data contained within the article are available upon request.

## Supplementary Materials

The following supporting information can be downloaded at https://www.mdpi.com/article/10.3390/molecules30010062/s1: Table S1: Properties of *E. invadens* PRMT genes and proteins; Table S2: Comparison between EiPRMTs and EhPRMTs.
