# Supplementary material for: PRMT5 Inhibitor EPZ015666 Decreases the Viability and Encystment of Entamoeba invadens
Source: Molecules. 2024 Dec 27;30(1):62. doi: 10.3390/molecules30010062 (PMC11721204; doi:10.3390/molecules30010062)
Supplement: Supplementary file 1 [file molecules-30-00062-s001.zip › molecules-3311236-supplementary.pdf]

Supplementary Table S1. Properties of *E. invadens* PRMT genes and proteins

Accession number	Gene length (bp)	Introns	mRNA length (bp)	Protein length (aa)	Molecular weight (kDa)	pI
**EIN_172090**	800[a]	NA[b]	NA	NA	NA	NA
**EIN_223690**	1978	2	1827	608	69.8	5.57
**EIN_398100**	1068	1	999	332	38.2	5.10
**EIN_497480**	966	No	966	321	37	5.22

[a] Incomplete gene, missing the 5′-end

[b] Not Applicable

Table S2. Comparison between EiPRMTs and EhPRMTs

	EhPRMT1a	EhPRMT1b	EhPRMT1c	EhPRMTA	EhPRMT5
**EIN_497480**	**57.55**[a]	42.95	41.01	29.64	17.18
**EIN_398100**	41.64	42.51	**83.73**[a]	24.53	19.40
**EIN_172090**	27.06	28.35	27.76	**58.17**[a]	15.77
**EIN_223690**	19.10	22.97	18.73	15.60	**46.32**[a]

[a] highest identity
